# Functional Fibronectin Adsorption on Aptamer-Doped Chitosan Modulates Cell Morphology by Integrin-Mediated Pathway

**DOI:** 10.3390/ma12050812

**Published:** 2019-03-08

**Authors:** Ludovica Parisi, Andrea Toffoli, Massimiliano G. Bianchi, Carlo Bergonzi, Annalisa Bianchera, Ruggero Bettini, Lisa Elviri, Guido M. Macaluso

**Affiliations:** 1 Centro Universitario di Odontoiatria, Università degli Studi di Parma, Via Gramsci 14, 43126 Parma, Italy; andrea.toffoli@unipr.it (A.T.); guidomaria.macaluso@unipr.it (G.M.M.); 2 Dipartimento di Medicina e Chirurgia, Università degli Studi di Parma, Via Gramsci 14, 43126 Parma, Italy; massimiliano.bianchi@unipr.it; 3 Dipartimento di Scienze degli Alimenti e del Farmaco, Università degli Studi di Parma, Parco Area delle Scienze 59/A, 43124 Parma, Italy; carlo.bergonzi@studenti.unipr.it (C.B.); annalisa.bianchera@unipr.it (A.B.); ruggero.bettini@unipr.it (R.B.); lisa.elviri@unipr.it (L.E.); 4 IMEM-CNR National Research Council, Parco Area delle Scienze 37/A, 43124 Parma, Italy

**Keywords:** DNA aptamers, biomaterials, fibronectin, integrins, cell morphology

## Abstract

A decisive step in cell-biomaterial interaction is represented by the adsorption of proteins at the interface, whose fine control may be useful to trigger proper cell response. To this purpose, we can selectively control protein adsorption on biomaterials by means of aptamers. Aptamers selected to recognize fibronectin dramatically enhance chitosan ability to promote cell proliferation and adhesion, but the underlying biological mechanism remains unknown. We supposed that aptamers contributed to ameliorate the adsorption of fibronectin in an advantageous geometrical conformation for cells, thus regulating their morphology by the proper activation of the integrin-mediated pathway. We investigated this possibility by culturing epithelial cells on chitosan enriched with increasing doses of aptamers in the presence or in the absence of cytoskeleton pharmacological inhibitors. Our results showed that aptamers control cell morphology in a dose dependent manner (*p* < 0.0001). Simultaneously, when the inhibition of actin polymerization was induced, the control of cell morphology was attenuated (*p* < 0.0001), while no differences were detected when cells contractility was challenged (*p* > 0.05). Altogether, our data provide evidence that aptamers contribute to control fibronectin adsorption on biomaterials by preserving its conformation and thus function. Furthermore, our work provides a new insight into a new way to accurately tailor material surface bioactivity.

## 1. Introduction

Biological tissues are complex systems and their structural and molecular organizations rely on their functions. As such, constructs for tissue engineering (TE), which are designed to restore, replace or regenerate lost damaged tissues, have to be accurately tuned in order to trigger an active dialogue with the surrounding biological milieu, most importantly cells, thus promoting proper healing and regeneration [1,2]. 

**Figure 1:**
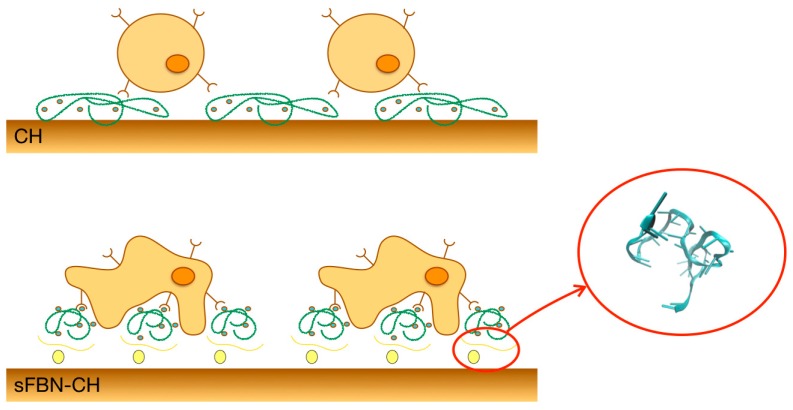
Diagram representing the rationale for the aptamer-coated scaffold to retain fibronectin in a suitable geometric conformation. Un-coated scaffold (CH) adsorbs fibronectin from the surrounding milieu, mostly inducing the masking of cell binding domains. Aptamers specifically lead the adsorption of fibronectin (sFBN-CH) and enrich scaffold with docking points for effective cell adhesion. The 3D rendering of the anti-fibronectin aptamer is a courtesy of Base Pair Biotechnologies (Pearland, TX, USA).

Chitosan is a polysaccharide derived from the partial de-acetylation of chitin, the main component of crustacean and arthropods exoskeleton. Chitin de-acetylation, which occurs through enzymatic or chemical hydrolysis under severe alkaline conditions, confers to chitosan the capacity to be easily protonated and unique structural versatility, thus making it an optimal candidate for TE scaffold fabrication [3,4]. Furthermore, it has been widely described that chitosan possesses high affinity for proteins, which are of the utmost importance in addressing cell response at the interface [4]. However, our previous experiences highlighted a low-supportive capacity of chitosan in term of cell colonization [5]. To justify the mismatch between high protein adsorption and poor cell response, according to Andrade et al. we previously hypothesized that the cationic properties of chitosan may induce a shift in protein conformation, that randomly determine which side of the molecule should interact with the material and which with the surrounding milieu, thus potentially influencing the exposure of responsive points for cells [6,7]. Therefore, the preservation of pristine protein conformation functionality at the interface of chitosan is desirable. To address this issue, we have recently conferred to chitosan a selective binding capacity, by means of aptamers (Figure 1) [8]. Aptamers are small oligonucleotides, which are able to recognize, bind and retain target molecules, including proteins, by assuming a high specific three-dimensional conformation [9].

In particularly, we have focused our previous efforts on fibronectin (FBN) [8]. FBN has been chosen as a target, because it is a protein abundant in wounded tissues and that plays a pivotal role in cell adhesion thank to the presence of cell binding domains within its structure, including the minimal recognition sequence for cell integrins (Arginine-Glycine-Aspartic Acid-RGD peptide) [10]. Integrins are a large family of homologous transmembrane receptors, constituted of two non-covalently associated glycoprotein subunits, alpha (α) and beta (β). After ECM molecules recognition (e.g., FBN), integrins are activated, α and β parts cluster and the cytoplasmic tail of the β subunit binds intracellular proteins that form mature focal adhesions, which interact with actin bundles to control cytoskeleton organization and thus cell shape [11]. The α5β1 integrin is the isoform known to bind FBN and it is further recognized that a minimal conformational change in FBN tertiary structure greatly diminishes the ability of α5β1 to bind it [12].

Selective FBN chitosan (sFBN-CH) was obtained dressing chitosan by means of aptamers selected for recognizing FBN. Aptamer-modification showed for the first time to dramatically improve murine osteoblasts (MC3T3-E1) colonization on chitosan, as to better preserve pristine FBN conformation once adsorbed on chitosan [8,13]. 

The aim of the present work is to confirm that the molecular mechanisms behind the amelioration of cell behavior at the interface of sFBN-CH are related to FBN functional adsorption, thus modulating cell shape and spreading by actin modulation. To this purpose, we studied the shape of cells on chitosan modified with increasing amounts of anti-FBN aptamers and under cytoskeleton modulation.

## 2. Materials and Methods 

### 2.1. Specimens

#### 2.1.1. Chitosan Preparation

Chitosan discs were prepared as previously described [8]. Briefly, a 2% chitosan solution was prepared by dissolving purified 90% de-acetylated chitosan powder (A.C.E.F., Piacenza, Italy) in a 1% acetic acid solution (Sigma-Aldrich, Saint Louis, MO, USA). D(+)-Raffinose (Sigma-Aldrich) was then added at a final concentration of 290 mM as viscosity modifying agent [14]. The one-milliliter solution was thus spread out onto a glass support to obtain a uniform film 0.25 mm thick and dried at 45 °C for 1 h in a ventilated oven. The film was thus transferred in a 5% potassium hydroxide (Sigma-Aldrich) gelation solution for 24 h and subsequently cut to discs fitting for 48-wells culturing plate.

Chitosan discs were rinsed twice in Phosphate Buffer Saline (PBS, Thermo Fisher Scientific, Waltham, MA, USA) and cleaned under UV light for 10 min.

#### 2.1.2. Anti-Fibronectin Aptamer

Single-stranded DNA-based aptamers screened against human and bovine FBN were used in this study (Base Pair Biotechnologies, Pearland, TX, USA). Aptamers were modified with a carbon chain containing a disulfide bond on their 3’-end and with a biotin on their 5’-end. 

Prior to chitosan functionalization, thiol group at the 3’-end was exposed by reducing the disulfide bond with aptamer reducing buffer (Base Pair Biotechnologies, Pearland, TX, USA) for 10 min. Excess of reducing agent was then removed with a chromatographic column (mini Quick Spin Oligo Columns, Roche Life Science, Branford, CT, USA) and aptamers were finally diluted at their working concentration in aptamer folding buffer (Base Pair Biotechnologies) [8,15].

#### 2.1.3. sFBN-CH and CH

The surface of chitosan discs was modified with anti-FBN aptamers by exploiting chitosan capacity to spontaneously adsorb sulfur-containing compounds [16]. Each chitosan disc was incubated with 100 µL of anti-FBN solution for 1 h (sFBN-CH), while unmodified chitosan (CH) was incubated 1 h with 100 µL of aptamer folding buffer and used as a control as previously described [8].

Four different anti-FBN aptamer concentrations were used for the test groups: 0.05, 0.1, 0.2 and 0.4 µg/µL. Previous studies have been conducted to assess the dose of aptamers [8].

### 2.2. Protein Adsorption Studies

#### 2.2.1. Bradford Assay

Bradford assay was used to study the time-course of blood plasma proteins and of pure FBN adsorption to chitosan with or without aptamers.

To this purpose, discs were soaked for 1 h in 200 µL of PBS supplemented with 2% of human serum (Pooled Normal Human Serum, Innovative Research, Peary Court, FL, USA) either of pure FBN (F0895, Sigma-Aldrich) at a concentration comparable with that present in a 2% human serum solution (0.02 mg/mL). Protein amount in supernatants was quantitated after 5, 15, 30, 45 and 60 min through Bradford assay (BIO-RAD Protein Assay, BIO-RAD, Hercules, CA, USA) according to the manufacturer’s recommendations. Ten-µl aliquots were collected at each time point, mixed with 200 µL of Bradford Working Solution and incubated at 37 °C for 2 min prior to absorbance assessment at 620 nm with a micro plate photometer (Multiskan™ FC, Thermo Fisher Scientific, Waltham, MA, USA). The number of proteins deposited on the discs was finally calculated by subtracting the residual concentration in supernatant from the initial one.

#### 2.2.2. Western Blot

WB was developed at the end of the time-course analysis to assess the selectivity of sFBN-CH for fibronectin.

Samples were briefly rinsed twice in PBS to remove softly bound proteins, incubated with 80 µL of Sample Buffer 1X (Tris-HCl 62.5 mM pH 6.8, SDS 1.5% w/v, DTT 100 mM) and then freeze, thawed sonicated for 15 min and boiled at 95 °C for 10 min to completely recover adsorbed proteins. Equal volumes of recovered proteins were loaded on a 12% polyacrylamide gel (Acrylamide/Bis-Acrylamide 30%, Sigma-Aldrich), electrophoresed 1 h at 180 V and subsequently blotted on a PVDF membrane (Immobilon-P, Darmstadt, Germany) 1 h at 100 V. Non-specific sites were firstly blocked 1 h at room temperature (RT) in Tris-buffered saline (TBS, Tris-HCl 50 mM pH 7.5 and NaCl 150 mM) containing 10% of blocking reagent (Roche S.p.A., Segrate, Italy). Then, the membrane was prior incubated overnight with an anti-FBN (F3648, Sigma-Aldrich) antibody diluted 1:800 in 0.1% Tween 20 in TBS supplemented with 5% bovine serum albumin (Sigma-Aldrich) and then with an HRP-conjugated secondary antibody (Cell Signalling Technology, Danvers, MA, USA) diluted 1:10,000. Eventually, immunoreactivity was visualized with enhanced chemiluminescence (Immobilon Western Chemiluminescent HRP, Sigma-Aldrich).

### 2.3. Cell Assays

#### 2.3.1. Cell Culture

In vitro assays were performed with human cervical cancer (HeLa) cell line (Sigma-Aldrich). 

Cells were cultured up to 4 days in 48-well plates at a density of 5000 cells/sample and maintained in complete high glucose Dulbecco modified Eagle’s medium (DMEM, Thermo Fisher Scientific) supplemented with 10% Fetal Bovine Serum (FBS, Thermo Fisher Scientific) and 1% Penicillin and Streptomycin (PenStrep, Thermo Fisher Scientific).

#### 2.3.2. Cytoskeleton Inhibitors Cytotoxicity Analysis

Pharmacological agents cytochalasin-D and blebbistatin were used to inhibit actin polymerization and myosin II contractility respectively. 

Experiments were undertaken to define the concentration that did not elicit signs of cytotoxicity and which allowed the spreading of the cells with no changes in their morphology (Figure S1). To this purpose, cells were treated with increasing amounts of cytochalasin-D (0.1, 0.25, 0.5 and 0.75 µM: C8273, Sigma-Aldrich) and blebbistatin (0.1, 0.5, 1 and 1.5 µM: 203390, Sigma-Aldrich) and monitored for viability and morphology up to 4 days.

Cell viability was assessed with chemiluminescence (CellTiterGLO, Promega, Milano, Italy). Briefly, samples were incubated with 100 µL of a 1:1 solution of culturing medium and lysis buffer for 2 min on an orbital shaker, thus stabilized at RT in dark conditions for 10 min. Eventually, samples were briefly spanned, and luminescence quantitated with a luminometer (GloMax® 20/20, Promega, Fitchburg, WI, USA).

Simultaneously, cell morphology was assessed by crystal violet (CV) staining, with an inverted microscope (Eclipse TS100, Nikon, Tokyo, Japan). To this purpose, cells were fixed 20 min at RT in paraformaldehyde 4%, thus stained with a 5 mg/mL solution of CV (491502, Carlo Erba, Cornaredo, Italy) prepared in 20% methanol (Sigma-Aldrich) for 10 min and rinsed twice in distilled water. When samples were completely dried, specimens were observed and microphotographs acquired with a digital camera (Digital Sight DS-Fi2, Nikon).

Cytochalasin 0.1 µM and blebbistatin 1 µM were finally chosen as cytoskeleton inhibitor concentrations for further experiments.

#### 2.3.3. Cell Morphology Analysis

To assess the effects of aptamers on cell shape, morphology and adhesion, cells were cultured up to 4 days on CH and sFBN-CH specimens at increasing aptamer concentrations and in the presence or in the absence of cytoskeleton inhibitors. The concentration of the latter substances reflected the previous analysis.

Cells were daily observed with an inverted microscope (Eclipse TS100, Nikon). Photographs were acquired with a digital camera (Digital Sight DS-Fi2, Nikon) and image analysis was performed with the D3 software (Version 4.3, Nikon). In particular, the amount of spread and non-spread cells on chitosan surface was measured after the region of interest identification (ROI). Cells were considered as a spread when philopodia were clearly visible and their circularity coefficient was lower than 0.8.

### 2.4. Statistical Analysis

Data were analyzed using Prism6 (GraphPad, La Jolla, CA, USA) and are reported as the mean ± SD of three repeated experiments performed in triplicates. Differences between groups were evaluated with two-way ANOVA statistical test combined to Sidak multiple comparisons post-hoc test and considered significant when *p* < 0.05. Trends were fitted with linear regression approximation with a 95% interval confidence.

## 3. Results

### 3.1. Anti-FBN Aptamers Interface Modification Induces Firm FBN Adsorption

**Figure 2:**
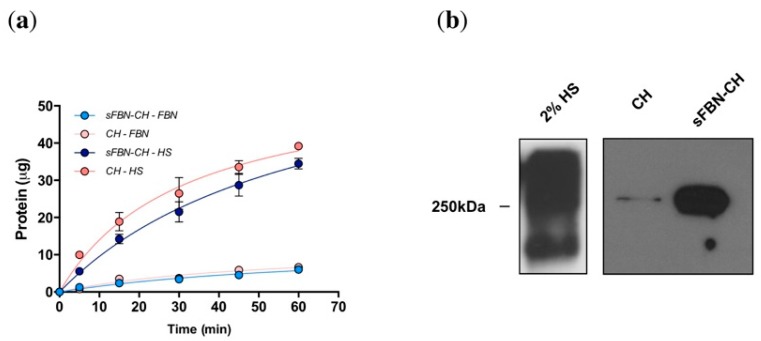
Protein adsorption over time and aptamer-doped chitosan selectivity for FBN. (**a**) Time-course of serum proteins and of pure FBN deposition on CH and sFBN-CH samples. (**b**) Western blot analysis of FBN stably adsorbed on CH and on sFBN-CH.

Serum proteins showed very fast deposition on chitosan both in the presence or in the absence of aptamer functionalization (Figure 2a). As a tendency, slightly more proteins seemed to be adsorbed on CH (39.2 ± 1.0 µg) versus sFBN-CH (34.5 ± 1.4 µg), even though no significant differences were revealed after the statistical analysis (*p* = 0.2034). The time-courses resulted comparable and estimated to hyperbolic trends (CH R^2^ = 0.9789; sFBN-CH R^2^ = 0.9866). Consistently with this, when CH or sFBN-CH specimens were incubated 1h with a solution of pure FBN at serum concentrations, no differences were revealed among the groups (CH 6.6 ± 0.1; sFBN-CH 6.0 ± 0.1 µg; CH vs. sFBN-CH *p* = 0.2352; CH R^2^ = 0.9547; sFBN-CH R^2^ = 0.9755).

Furthermore, to investigate whether aptamers enhanced the firm adsorption of FBN a WB analysis was performed. Figure 2b shows that chitosan selectivity for FBN was 34.7-fold promoted by aptamers (O.D. CH = 2.8% vs. O.D. sFBN-CH = 97.2%). 

All together, these data indicate that aptamers promote a more fixed adsorption of FBN on the surface.

### 3.2. Anti-FBN Aptamers Interface Modification Promotes Epithelial Cells Adhesion in A Dose-Dependent Manner

To investigate if aptamers improve the adhesion of cells to chitosan, the number of flattened cells was monitored over the time up to day 4 and quantitated by image analysis (Representative cell images are reported in Supplementary Materials—Figure S2).

**Figure 3:**
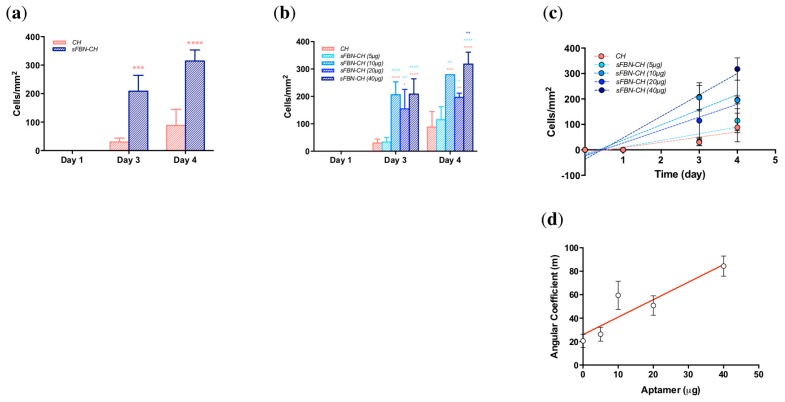
HeLa cells spreading on sFBN-CH. (**a**) Histograms showing the number of spread cells on CH and sFBN-CH after 1, 3 and 4 days of culture. (***b***) Histograms the number of spread cells on CH and sFBN-CH enriched with increasing doses of aptamers after 1, 3 and 4 days of culture. (**c**) The trend of cell spread rate on chitosan discs implemented with increasing aptamer doses. (**d**) The trend of angular coefficients derived from cell spread on chitosan discs implemented with increasing aptamer doses (* *=* *p* *<* 0.05).

The presence of aptamer dramatically increased the entity of cell spreading starting from day 3 (Figure 3a). After 1 day of culture, no spread cells were found both on CH and sFBN-CH samples, as well as no significant differences were detectable (*p* > 0.9999). However, 6.93-fold more at day 3 and 3.56-fold more cells at day 4 were spread on sFBN-CH, with statistically significant differences (day 3: CH vs. sFBN-CH *p* = 0.0002; day 4: CH vs. sFBN-CH *p* < 0.0001). 

Additionally, when different doses of aptamers were used, the amount of well-spread cells increased proportionally with the amount of total aptamer used, following linear regression trends (Figure 3b,c—CH R^2^ = 0.5723; sFBN-CH (5 µg) R^2^ = 0.6621; sFBN-CH (10 µg) R^2^ = 0.7529; sFBN-CH (20 µg) R^2^ = 0.7916; sFBN-CH (40 µg) R^2^ = 0.9068). After 3 days the differences with the control were significant when high doses of aptamers were used (CH vs. sFBN-CH (10 µg) *p* < 0.0001; CH vs. sFBN-CH (20 µg) *p* = 0.0036; CH vs. sFBN-CH (40 µg) *p* < 0.0001), as well as at day 4 (CH vs. sFBN-CH (10 µg) *p* = 0.0004; CH vs. sFBN-CH (20 µg) *p* = 0.0047; CH vs. sFBN-CH (40 µg) *p* < 0.0001). On the contrary, the minimum dose of aptamer used has never induced any significant change in cell morphology (CH vs. sFBN-CH (5 µg)-day 3 *p* > 0.9999; day 4 *p* = 0.8699). In addition to this, differences were detected also against the minimum amount of aptamer used and the other groups both at day 3 (sFBN-CH (5 µg) vs. sFBN.CH (10 µg) *p* < 0.0001; sFBN-CH (5 µg) vs. sFBN.CH (20 µg) *p* = 0.0048; sFBN-CH (5 µg) vs. sFBN.CH (40 µg) *p* < 0.0001) and at day 4 (sFBN-CH (5 µg) vs. sFBN.CH (10 µg) *p* = 0.0022; sFBN-CH (5 µg) vs. sFBN.CH (20 µg) *p* = 0.0458; sFBN-CH (5 µg) vs. sFBN.CH (40 µg) *p* < 0.0001). Furthermore, at day 4 the maximum amount of aptamer used significantly enhance the cell spreading if compared to low amounts (sFBN-CH (20 µg) vs. sFBN-CH (40 µg) *p* = 0.0014). 

The plot of the angular coefficients of the regression lines fitting cells spreading data with different amounts of aptamers revealed a linear regression trend with an estimated R^2^ value of 0.8322 (Figure 3d).

### 3.3. Integrin-Mediated Pathway Controls Epithelial Cells Adhesion at the Anti-FBN Aptamers Modified Interface

**Figure 4:**
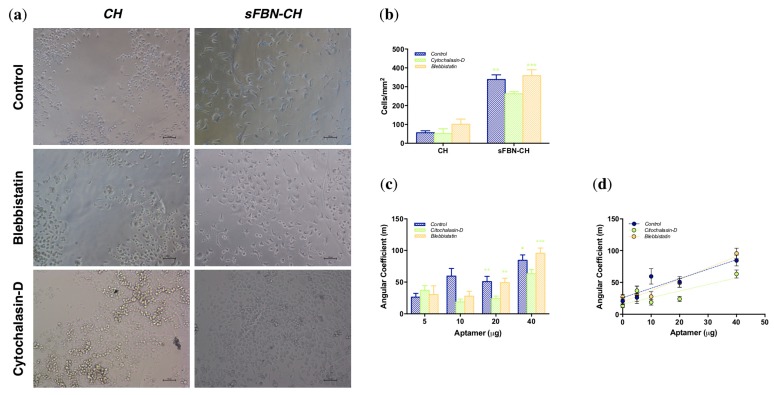
HeLa cells spreading modulation on sFBN-CH by cytochalasin-D and blebbistatin. (**a**) Representative images acquired with an inverted microscope of cells on CH and sFBN-CH specimens after 4 days of culture in complete medium or in complete medium supplemented with cytochalasin-D 0.1 µM and blebbistatin 1 µM. The magnification in Figure 4a is 10×. (**b**) Histograms showing the number of spread cells on CH and sFBN-CH after 4 days of culture in complete medium or in complete medium supplemented with cytochalasin-D 0.1 µM and blebbistatin 1 µM. (**c**) Histograms reporting angular coefficients derived from cell spread on chitosan discs implemented with increasing aptamer doses and cultured in complete medium or in complete medium supplemented with cytochalasin-D 0.1 µM or blebbistatin 1 µM. (**d**) The trend of angular coefficients derived from cell spread on chitosan discs implemented with increasing aptamer doses and cultured in complete medium or in complete medium supplemented with cytochalasin-D 0.1 µM and blebbistatin 1 µM (* *=* *p* *<* 0.05).

No differences in morphology of cells exposed to cytochalasin-D or blebbistatin and in untreated cells were detected on bare chitosan (Figure 4a,b). However, when actin polymerization was inhibited with cytochalasin-D on sFBN-CH, we observed a significant 1.29-fold or 1.37-fold reduction in the number of spread cells, when compared to untreated cells (*p* = 0.0043) and to cells cultured with blebbistatin (*p* = 0.0007), respectively. Interestingly, these two conditions did not show the difference between each other (*p* = 0.5496) (Figure 4a,b).

Furthermore, when the angular coefficients of linear regression trends derived from the use of different aptamer doses were compared (Figure 4c,d), a dip of the fitting curve was detected for cytochalasin-D treated group with significant differences to control and blebbistatin group when 20 or 40 µg of aptamers were applied (sFBN-CH (20 µg) control vs. cytochalasin-D *p* = 0.0025, blebbistatin vs. cytochalasin-D *p* = 0.0052; sFBN-CH (40 µg) control vs. cytochalasin-D *p* = 0.0279, blebbistatin vs. cytochalasin-D *p* = 0.0002). On the other hand, strict overlapping of control and blebbistatin group fitting lines was observed.

## 4. Discussion 

Protein adsorption on biomaterials occurs shortly after scaffold insertion and anticipates cells attachment. Therefore, the control of this phenomenon at the interface is extremely important when seeking to pilot cells fate [17]. Chitosan has been widely recognized as a suitable biomaterial for scaffold production, even if it possesses a scarce ability to support the attachment of mesenchymal, epithelial and osteoblast-like cells. Hence, the presence in the literature of different methods to enhance cell response on chitosan through FBN enrichment [8,18,19,20,21,22]. FBN control cell adhesion, spreading and proliferation on substrates through integrin binding, which is extremely dependant on FBN structural conformation. For examples, the α5β1 integrin recognizes FBN through the interaction with the RGD peptide, which is contained in the 10th type III repeat of FBN structure and that synergizes with a further peptide sequence (proline-histidine-serine-arginine-aspargine—PHSRN peptide) contained in the adjacent 9th type III domain of FBN. In bulk conditions, these two peptides are separated from 32 Å and this distance results to be extremely important for specific recognition between FBN and α5β1 integrin. It has been indeed demonstrated that a 23 Å removal greatly diminishes the binding capacity among FBN and α5β1 integrin [12]. Therefore, the control of FBN adsorption on biomaterials is important both from a quantitative and qualitative point of view.

In our previous work we proposed a novel method to ameliorate the adsorption of autologous FBN on chitosan through the use of selective binding molecules: Aptamers. We obtained an sFBN-CH that did not influence the amount of FBN adsorbed at the interface, but which induced an impressive amelioration of osteoblasts proliferation, adhesion and morphology [8]. As such, we hypothesized a potential role of aptamers in controlling FBN conformational adsorption. We confirmed this finding in the present experiments (Figure 2a), even though after two rinses in PBS, WB analysis revealed a higher signal of FBN adsorbed on sFBN-CH, showing a much stronger and stable adsorption pattern. Interestingly, these data comply with the results we obtained in a previous work testing a selective FBN hyaluronic acid (sFBN-HA) [15]. The use of anti-FBN aptamers appears therefore a valid general system to obtain materials capable to stably adsorb FBN. However, our recent findings have also confirmed our hypothesis of a different biological activity of FBN once adsorbed on CH or on sFBN-CH [13]. As such, the role of aptamers on chitosan seems to be double and the aim of the present work was thus that of confirming this finding supports the hypothesis of amelioration of FBN activity with biological data.

Our experiments showed striking effects of sFBN-CH on human epithelial cells adhesion. Chitosan modified with the highest dose of anti-FBN aptamers (40 µg) dramatically increased the number of well-spread cells with a pancake-like shape on day 3 and 4 (Figure 3a). Furthermore, when increasing the amount of anti-FBN aptamers (0, 5, 10, 20 and 40 µg), the rate of cell spreading was progressively enhanced, suggesting an arising presence of functional FBN-related binding domains for cells at the interface (Figure 3b–d). Consistently with this, when cells were cultured in the presence of actin polymerization inhibitor cytochalasin-D, the rate of cell spreading decreased (Figure 4). Interestingly, the effects of cytochalasin-D were more substantial when sFBN-CH with higher doses of anti-FBN aptamers were used. Additionally, when myosin II contractility was inhibited, no significant differences in cell shape were observed. Since myosin II contractility does not directly depend on integrin activation, which is in turn regulated by the proper exposition of FBN RGD binding-motif, these data support the hypothesis that sFBN-CH induces an FBN adsorption pattern that is fully functional to eventually influence actin bundles reorganization and cell shape. It should be stressed that cells were treated with cytoskeleton inhibitors concentrations that did not elicit signs of cytotoxicity and which allowed the complete spreading of the cells on the tissue culture plate (TCPs) with no changes of morphology as reported in the material and methods section. Therefore, any variance of cell morphology can be solely attributed to the functional adsorption of FBN on sFBN-CH.

The hypothesis of an FBN functional adsorption on chitosan is further supported by the literature. For example, observing the preferential adhesion of valve endothelial cells (VEC) on TCPs covered with increasing doses of FBN, Cuy et al. tried to ameliorate chitosan bioactivity by an FBN coating [22]. Surprisingly, VEC morphology on FBN-coated chitosan was not different from the control and their growth was lower than the growth on TCPs. Noteworthy, no differences were detected between the amount of FBN adsorbed on TCPs and on chitosan, suggesting a loss in bioactivity of FBN when adsorbed on chitosan with the respect of TCPs. Consistently with this Custodio et al. observed that when FBN was covalently immobilized on chitosan via carbodiimide chemistry, the adhesion of SaOS-2 cells was promoted if compared to FBN spontaneous adsorption, confirming the hypothesis of a probable loss in bioactivity when FBN is simply adsorbed on chitosan [21].

To conclude, we demonstrated that sFBN-CH was able to dramatically increase the number of adhering cells. A similar modified hyaluronic acid-based hydrogel (sFBN-HA) [16] showed comparable effects, but, interestingly, different mechanisms appear to be involved. While sFBN-HA quantitatively increased the adsorption of FBN, enhancing cells adhesion, sFBN-CH showed a similar amount of adsorbed FBN when compared to the control CH, but with great differences in the strength and functionality of the adsorption, as discussed above. Consistent with these findings, additional studies that aimed at directly investigating the structural conformation of FBN on CH and on sFBN-CH have confirmed the role of function-wise conformational differences of adsorbed FBN [13].

## Supplementary Materials

The following are available online at https://www.mdpi.com/1996-1944/12/5/812/s1, Figure S1: Cytochalasin-D and blebbistatin cytotoxicity on human HeLa cells. Figure S2: HeLa cells spreading on CH and on sFBN-CH.
