# Supplementary material for: Functional Fibronectin Adsorption on Aptamer-Doped Chitosan Modulates Cell Morphology by Integrin-Mediated Pathway
Source: Materials (Basel). 2019 Mar 8;12(5):812. doi: 10.3390/ma12050812 (PMC6427328; doi:10.3390/ma12050812)
Supplement: Supplementary file 1 [file materials-12-00812-s001.pdf]

materials

Supplementary Files

Functional Fibronectin Adsorption on Aptamer-Doped Chitosan Modulates Cell Morphology by Integrin-Mediated Pathway

Ludovica Parisi [1,2,*], Andrea Toffoli [1,2], Massimiliano G. Bianchi [2], Carlo Bergonzi [3], Annalisa Bianchera [3], Ruggero Bettini [3], Lisa Elviri [3] and Guido M. Macaluso [1,2,4]

1 Centro Universitario di Odontoiatria, Università degli Studi di Parma, Via Gramsci 14, 43126 Parma, Italy; andrea.toffoli@unipr.it (A.T.); guidomaria.macaluso@unipr.it (G.M.M.)
2 Dipartimento di Medicina e Chirurgia, Università degli Studi di Parma, Via Gramsci 14, 43126 Parma, Italy; massimiliano.bianchi@unipr.it
3 Dipartimento di Scienze degli Alimenti e del Farmaco, Università degli Studi di Parma, Parco Area delle Scienze 59/A, 43124 Parma, Italy; carlo.bergonzi@studenti.unipr.it (C.B.); annalisa.bianchera@unipr.it (A.B.); ruggero.bettini@unipr.it (R.B.); lisa.elviri@unipr.it (L.E.)
4 IMEM-CNR National Research Council, Parco Area delle Scienze 37/A, 43124 Parma, Italy
* Correspondence: ludovica.parisi@unipr.it; Tel.: +39-0521-906742

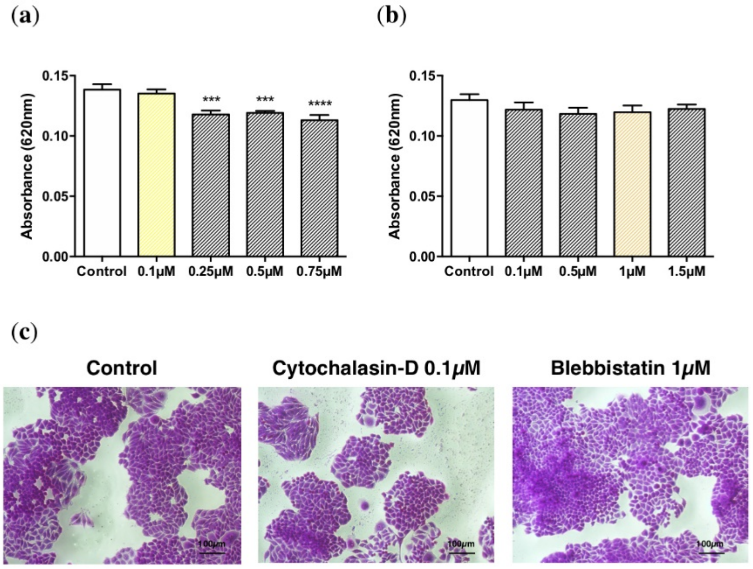

Figure S1. Cytochalasin-D and blebbistatin cytotoxicity on human HeLa cells. (a) Cell viability of HeLa cells up to 4 days treating with cytochalasin-D at increasing concentrations. (b) Cell viability of HeLa cells up to 4 days treating with blebbistatin at increasing concentrations. (c) Representative images acquired with an inverted microscope of HeLa cells stained with CV after 4 days of culture in complete medium or in complete medium supplemented with cytochalasin-D 0.1µM and blebbistatin 1 µM. (* = $p < 0.05$). Cytochalasin-D showed a negative effect on cell viability, which was significantly reduced at concentrations higher than 0.1 µM (Day 4: Control vs. 0.25 µM $p = 0.0002$; Control vs. 0.5 µM $p = 0.0003$; Control vs. 0.75µM $p < 0.0001$). Consistently, after CV staining cell morphology was severely affected by cytochalasin-D. With increasing amount of cytochalasin-D used, cells appeared more elongated, they lost contacts and adhesion among them when organized in cluster and finally they fused forming structures like syncytiums. On the counterpart, blebbistatin did not show negative effects on cell viability up to 1.5 µM. However, cell morphology was affected when doses higher than 1 µM were applied.

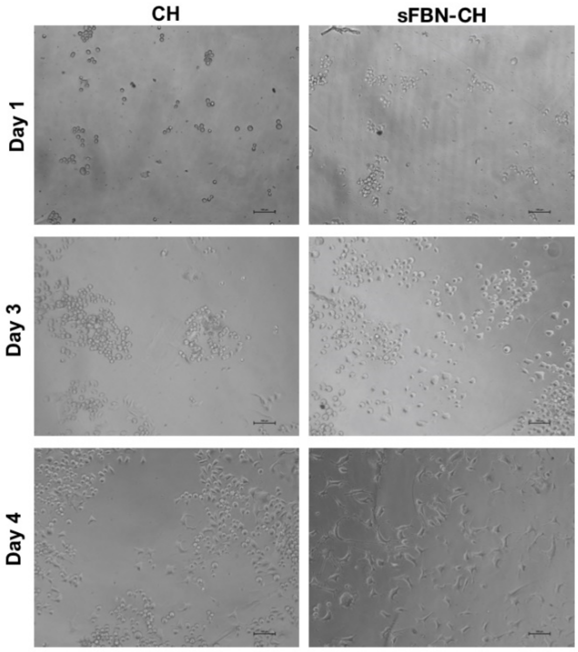

Figure S2. HeLa cells spreading on CH and on sFBN-CH. Representative images acquired with an inverted microscope of cells on CH and sFBN-CH specimens after 1, 3 and 4 days of culture.

© 2019 by the authors. Submitted for possible open access publication under the terms and conditions of the Creative Commons Attribution (CC BY) license (http://creativecommons.org/licenses/by/4.0/).
